# Efficacy of Treatment with the Antibiotic Novobiocin against Infection with *Bacillus anthracis* or *Burkholderia pseudomallei*

**DOI:** 10.3390/antibiotics11121685

**Published:** 2022-11-23

**Authors:** Christopher P. Klimko, Susan L. Welkos, Jennifer L. Shoe, Sherry Mou, Melissa Hunter, Nathaniel O. Rill, David DeShazer, Christopher K. Cote

**Affiliations:** Bacteriology Division, United States Army Medical Research Institute of Infectious Diseases (USAMRIID), Fort Detrick, MD 21702, USA

**Keywords:** *Burkholderia pseudomallei*, *Bacillus anthracis*, novobiocin, early-generation antibiotics, broad spectrum, biothreats, mice, melioidosis, anthrax

## Abstract

The microbial pathogens *Burkholderia pseudomallei* and *Bacillus anthracis* are unrelated bacteria, yet both are the etiologic agents of naturally occurring diseases in animals and humans and are classified as Tier 1 potential biothreat agents. *B. pseudomallei* is the gram-negative bacterial agent of melioidosis, a major cause of sepsis and mortality globally in endemic tropical and subtropical regions. *B. anthracis* is the gram-positive spore-forming bacterium that causes anthrax. Infections acquired by inhalation of these pathogens are challenging to detect early while the prognosis is best; and they possess innate multiple antibiotic resistance or are amenable to engineered resistance. Previous studies showed that the early generation, rarely used aminocoumarin novobiocin was very effective in vitro against a range of highly disparate biothreat agents. The objective of the current research was to begin to characterize the therapeutic efficacy of novobiocin in mouse models of anthrax and melioidosis. The antibiotic was highly efficacious against infections by both pathogens, especially *B. pseudomallei*. Our results supported the concept that specific older generation antimicrobials can be effective countermeasures against infection by bacterial biothreat agents. Finally, novobiocin was shown to be a potential candidate for inclusion in a combined pre-exposure vaccination and post-exposure treatment strategy designed to target bacterial pathogens refractory to a single medical countermeasure.

## 1. Introduction

*Burkholderia pseudomallei* is a gram-negative pathogen and agent of melioidosis, a disease with forms ranging from acute and rapidly fatal to protracted and chronic. It is a major cause of sepsis and mortality globally in endemic tropical and subtropical regions [1,2]. *Bacillus anthracis*, a gram-positive spore-forming bacterium, is the etiologic agent of anthrax [3,4,5]. Besides causing natural infections, both organisms are biothreat agents and are classified as Tier 1 Select Agents by the U.S. Department of Health and Human Services [6]. Both bacteria are highly infectious, notably via inhalation, and exposures by the aerosol route exhibit nonspecific early signs of infection. Thus, early diagnosis of these diseases can be very challenging and unless there is a high index of suspicion, the diseases can rapidly progress to a stage at which they are no longer treatable (*B. anthracis*) or requires protracted therapy (*B. pseudomallei*) with antibiotics. Finally, antibiotic treatment of these infections can fail due to innate multiple drug resistance (*B. pseudomallei*) or potentially engineered resistance (either bacterium). 

The objective of our research was to repurpose and evaluate the antimicrobial agent novobiocin (Novo), an aminocoumarin class antibiotic, as a novel therapeutic approach to counteract natural or engineered resistance to conventional antibiotics, promote more complete and rapid clearance of infection, and increase survival of mice exposed to a lethal infection with the bacterial threat agents of melioidosis and anthrax. Novo is a potent bactericidal antibiotic having broad in vitro efficacy against several high consequence pathogens [7,8,9]. It is a licensed but under-utilized early-generation antibiotic which exhibits low effective concentrations against highly disparate agents such as *Francisella tularensis*, the pathogenic *Burkholderia* spp., and *Bacillus anthracis* [8,9,10,11,12]. The minimum inhibitory concentration (MIC) of Novo for *B. anthracis* and *B. pseudomallei* were determined to be 0.82 (range 0.49–1.95) µg/ mL and 1.63 (range 0.98–1.95) µg/mL respectively [8]. Novo is potentially particularly useful for the treatment of melioidosis due to the natural resistance of *B. pseudomallei* to many antibiotics, to include strains exhibiting resistance to the current drugs of choice, as exemplified in recent reports [13,14,15]. 

Furthermore, we decided to use relatively short treatment regimens of Novo, a decision based on the goal of identifying treatments for anthrax and melioidosis that are effective, more practical to administer in the field (in both public health and biothreat scenarios) than current treatment options, and more cost-effective. Post-exposure prophylaxis for anthrax can be up to 60 days, although shorter regimens have been described [16,17,18,19], and treatment for *B. pseudomallei* is complex and includes a minimum 14 day intensive phase and 3 to 6 month eradication phase [20,21,22,23]. More stringent treatment conditions were utilized in the current work to provide a proof-of-concept justifying further optimization of Novo as a treatment option.

## 2. Results

### 2.1. Antibiotic Efficacy against Parenteral Challenge with B. anthracis or B. pseudomallei

In each of the antibiotic efficacy studies to be presented, the dose and time of Novo administration relative to the infection are detailed in the figure legends. 

#### 2.1.1. Anthrax Model

**Figure 1:**
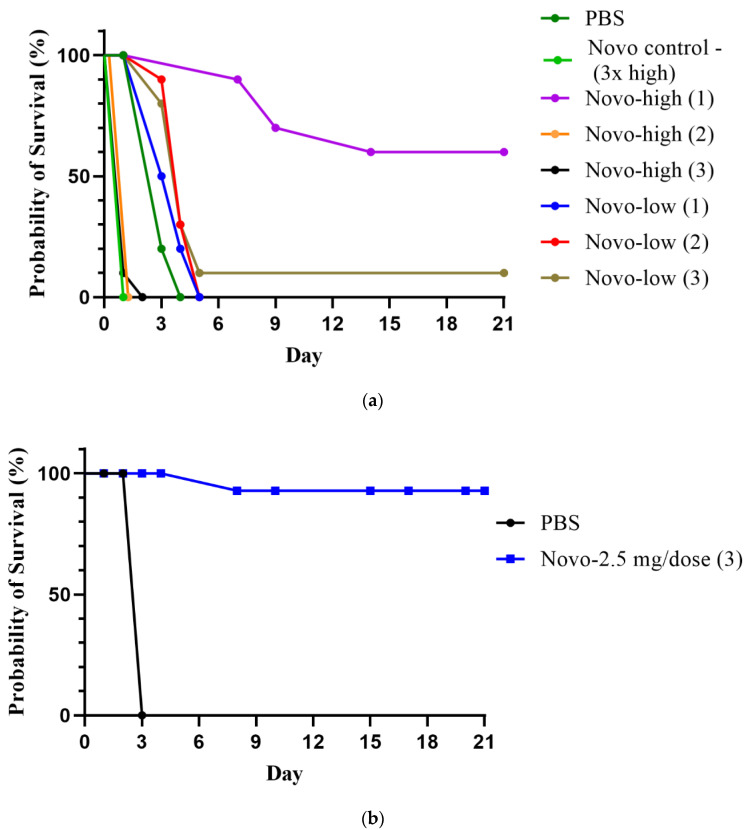
A/J mice infected via subcutaneous (SC) injection with *B. anthracis* Sterne spores and treated with Novo. (**a**) Mice were challenged with approximately 1.6 × 10^4^ spores of *B. anthracis* Sterne (15 LD_50_), and treatments consisted of a high (5.0 mg) or low (1.25 mg) dose of Novo administered via intraperitoneal [IP] injection. One, 2, or 3 doses of Novo were given post-challenge (1 h, 1 h and 8 h, or 1 h, 8 h, and 24 h). Mice were monitored for 21 days. (**b**) A second iteration of Novo with a potentially optimal dosing scheme. Mice were challenged SC with 1.1 × 10^4^ spores of *B. anthracis* Sterne (10 LD_50_) and treated with Novo (2.5 mg) at 1 h, 8 h, and 24 h post-challenge. All groups had 10 mice.

We evaluated the effects of antimicrobial treatments on the survival of A/J mice that had been infected with *B. anthracis* strain Sterne spores by the subcutaneous (SC) route. The results are presented in Figure 1a. Kaplan-Meier survival analysis showed that the survival curves were significantly different overall, *p* < 0.0001. The high dose of Novo (5 mg) was toxic if given more than once (100% lethal after 2 or 3 doses), but one dose was nontoxic and 60% protective (*p* = 0.0108 vs. all other infected groups). The low concentration of Novo (1.25 mg) was nontoxic at all doses, but 3 doses only protected 10% of the mice from *B. anthracis* Sterne strain spores. However, the mice given 2 or 3 doses of 1.25 mg Novo had extended time-to-mortality (TTM) compared to the PBS control, *p* = 0.0017 and 0.0056, respectively (Figure 1a). The results of a second iteration of the experiment using an optimized Novo dosing scheme are shown in Figure 1b. Three doses of 2.5 mg Novo protected 90% of the mice; and all mice survived until the study endpoint. The survival rates at 7 and 21 days, and the TTM (days) of the Novo treated mice, were significantly greater than the PBS-treated control mice (*p* < 0.0001). 

#### 2.1.2. Melioidosis Model 

**Figure 2:**
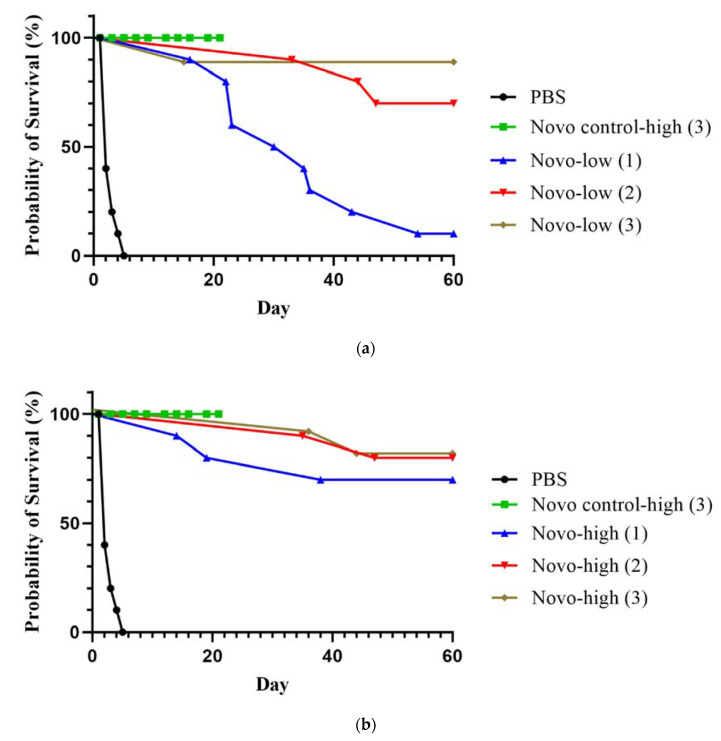
BALB/c mice infected by the IP route with *B. pseudomallei* and treated with Novo. The mice were challenged with approximately 2.0 × 10^5^ CFU *Bp* K96243 (5.7 LD_50_). One, 2, or 3 doses of Novo were given post-challenge (1 h, 1 h and 8 h, or 1 h, 8 h, and 24 h). The survival results are shown for animals given a low dose of Novo, 1.25 mg (**a**), or a higher dose, 2.5 mg (**b**). Survival was monitored for 60 days. All groups contained 10 mice.

Similar antibiotic efficacy studies were performed using a BALB/c mouse model of infection with *B. pseudomallei*. As shown in Figure 2, different Novo doses and administration schemes were evaluated in mice infected by the IP route with a lethal dose of *B. pseudomallei*. Three doses of 2.5 mg of Novo did not result in any toxicity in control mice that were monitored for 21 days. Survival was monitored for 60 days post-challenge, and the rates were significantly different overall by log-rank analysis, *p* < 0.0001. All Novo treatments except the single low dose (1.25 mg) were protective (70–90%), as shown in Figure 2a (low dose Novo) and Figure 2b (high dose Novo). Three doses of 1.25 mg Novo were 90% protective. This was significantly greater than the survival rate of mice receiving a single low dose of Novo (*p* = 0.0011), though not significantly different from the survival rates of mice receiving 2 low doses, or of mice treated 1 to 3 times with high dose Novo (2.5 mg). Mice in all Novo treatment groups had significantly extended TTM compared to the PBS control (*p* < 0.0001). 

The mice that were infected and not treated exhibited an acute course of rapidly fatal infection, like that observed previously for BALB/c mice, as illustrated in Figure 2 [24,25]. In contrast, the mice treated with one, 1.25 mg dose of Novo displayed typical signs of a chronic *B. pseudomallei* infection, the extent of which sometimes warranted early endpoint euthanasia in accordance with approved euthanasia intervention criteria. These signs included the formation of pyogranulomas at multiple sites in the body, circulatory problems such as lesion formation and necrosis of the tail, and rear-leg ataxia or paralysis (normally associated with pyogranulomatous inflammation in skeletal muscle, bone, or peripheral nerves in the hind limb), as described in detail elsewhere [24,25,26]. Such chronic infections are characteristic of the disease course of the more resistant C57BL/6 strain of mice [25,27,28,29,30]. The mice that were treated with 2 or 3 doses of Novo and survived lethal challenge showed no significant outward signs of clinical disease (Figure 2).

The extent of clearance of the *B. pseudomallei* K96243 challenge strain in survivors 60 days after challenge was investigated. Spleens were harvested from all survivors and bacterial burden was determined by enumeration of colony forming units (CFU) on sheep blood agar plates. As shown in Table 1, the short-course of antibiotics delivered soon after infection resulted in most survivors having no detectable bacterial burden in the spleen. *B. pseudomallei* has a well characterized ability to produce chronic infections that can be difficult to treat; and the spleen is an appropriate representative organ to gauge the extent of sterile immunity after vaccination or treatment [31,32,33,34,35]. However, the possibility exists that reservoirs of bacteria could be present in other tissues, that the bacteria entered a latent or non-culturable state, or that CFU were present below the limit of detection.

### 2.2. Antibiotic Efficacy against Inhalational Exposure in Models of B. anthracis and B. pseudomallei

#### 2.2.1. Anthrax Inhalational Model

**Figure 3:**
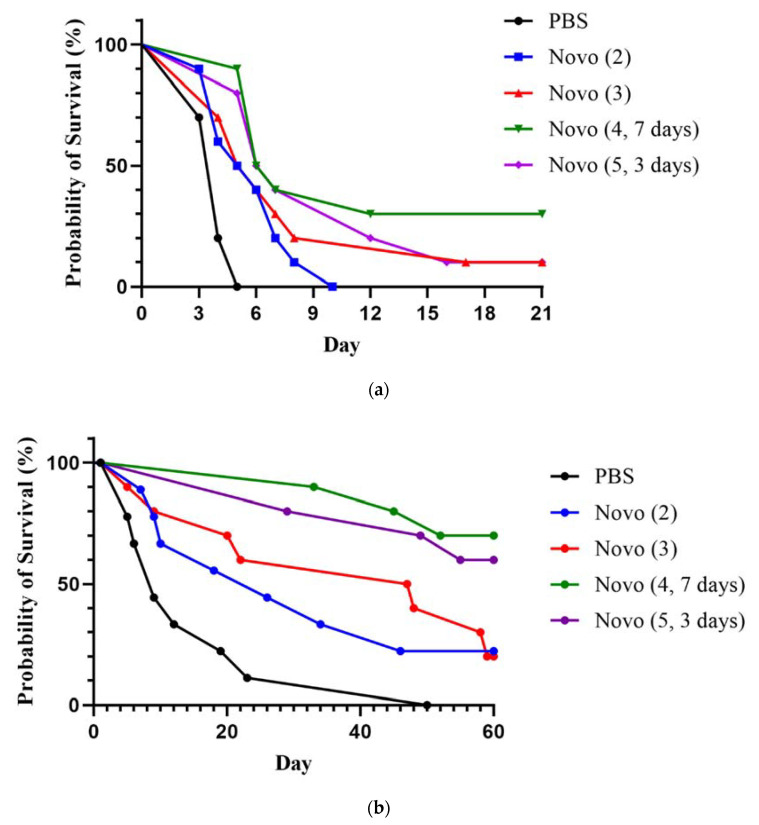
The effects of Novo treatment on mice infected with *B. anthracis* or *B. pseudomallei* by an inhalational route. (**a**) A/J mice (*n* = 10/group) were challenged intranasally with 8.6 × 10^5^ spores of *B. anthracis* Sterne (15 LD_50_) and treated with doses of 2.5 mg of Novo at the following times post-challenge: 1 h and 8 h (2-dose Novo); 1 h, 8 h, and 24 h (3-dose Novo); 1 h, 8 h, 24 h, 48 h, and 72 h (5 doses over 3 days Novo); or 1 h, 8 h, 24 h, and 168 h (4 doses over 7 days Novo). Survival was monitored for 21 days. (**b**) BALB/c mice were exposed by the aerosol route to approximately 43 CFU of *B. pseudomallei* K96243 (2 LD_50_) [25,27], and treated with the same dosing scheme as described in panel (**a**), and survival was monitored for 60 days. All groups contained 10 mice each except for the PBS and 2-dose Novo groups (in panel b) each of which had 9 mice.

We used the A/J mouse intranasal infection model to evaluate protection afforded against inhalational anthrax. The mice received approximately 8.6 × 10^5^ spores via intranasal instillation followed by Novo delivered via IP injection. As shown in Figure 3a, there were no significant differences in survival rates between treatment groups by day 7 or day 21 post-challenge (study endpoint). However, the greater extent of survival at day 7 of two treatment groups, the 4-dose (over 7 days) and 5-dose (over 3 days) mice, compared to the untreated PBS control mice suggested early Novo-associated protection (*p* = 0.087). Whereas four mice in each of these two treatment groups had survived by day 7, none of the ten PBS mice remained. Furthermore, mice in all four treatment groups (i.e., which received 2, 3, 4, or 5 doses of Novo) had significantly prolonged survival times compared to untreated mice. The TTM for the controls was 3.9 days, compared to 5.8 days (2-dose Novo, *p* = 0.0036), 6.7 days (3-dose Novo, *p* = 0.0133), and 6.9 days (5 doses over 3 days, *p* < 0.0001) groups. The 4-dose (over 7 days) treated group exhibited the longest TTM (8.3 days), *p* < 0.0001 compared to controls. Although the treatments did not significantly augment survival rates, given the extreme sensitivity to *B. anthracis* of mice by pulmonary routes, and the high intranasal challenge dose of Sterne used, the prolonged survival associated with Novo treatment was encouraging [36]. 

#### 2.2.2. Melioidosis Inhalational Model

Melioidosis is frequently acquired by inhalation [37,38]. BALB/c mice were exposed to aerosolized *B. pseudomallei* K96243 and treated with Novo. The dosing schedule for mice treated after aerosol exposure to approximately 43 CFU (2 LD_50_) is detailed in the legend for Figure 3b. By the study endpoint (60 days post-infection), treatment with 4 doses spanning 7 days had protected 70% of the mice, 5 doses given over 3 days yielded a 60% survival rate, and treatments with 2 or 3 doses of Novo protected 22% and 20% respectively; none of the PBS control mice survived (Figure 3b). The survival rates of both the 4-dose Novo and 5-dose Novo groups were significantly greater than that of the PBS controls (*p* = 0.011 or *p* = 0.003, respectively). In addition, by 21 days after treatments were completed, survival rates of both the 4-dose Novo and 5-dose Novo groups were significantly greater than those of the PBS control and 2-dose Novo groups, *p* = 0.0007 and *p* = 0.325, respectively). The TTM values (days) also differed and were 15.3 (PBS), 21.4 (2-dose Novo), 33.5 (3-dose Novo), 40.5 (5 doses over 3 days Novo), and 43.3 (4 doses over 7 days Novo). The TTM of the 4-dose Novo extended treatment group was significantly longer than that of all groups except for the 5-dose group, with *p* values from < 0.0001 (vs. PBS) to *p* = 0.026 (vs. 3x Novo). The TTM of the 5-dose group was also significantly or nearly greater than that of the mice receiving only 2 doses (*p* < 0.0001) or 3 doses (*p* = 0.0587). These results suggested that both the number of doses (a minimum of 4 doses of 2.5 mg Novo of), and the timing of their administration, impact the therapeutic efficacy of Novo against inhalational infection by *B. pseudomallei* K96243.

The organ CFU burden of mice surviving to 60 days after aerosol challenge with *B. pseudomallei* K96243 was determined on day 61 for lung and spleen samples, as described in the IP challenge experiment. One mouse receiving 4 doses of Novo did not show any clinical signs on day 60 but was found dead in the cage on day 61. Spleen and lung samples were collected from the remaining 16 survivors from the four groups of treated mice. No bacteria were recovered from any of the survivors’ tissues except for the spleen from one mouse in the 3-dose Novo group which had a visible pyogranuloma formation and a bacterial burden of approximately 1.2 × 10^8^ CFU/g. 

### 2.3. Novobiocin in Combination with a Representative Partially Protective Vaccine 

**Figure 4:**
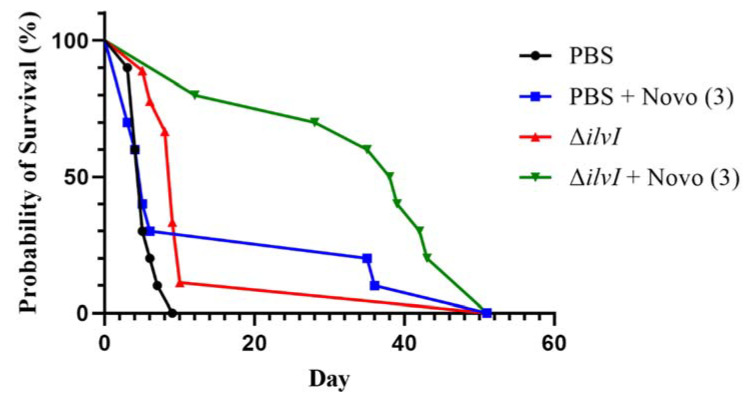
The protective effects for BALB/c mice exposed to aerosolized *B. pseudomallei* by vaccination with a live *Burkholderia* vaccine strain and treatment with Novo. The mice were vaccinated with the live attenuated *B. thailandensis* E555 Δ*ilvI* strain, exposed to *B. pseudomallei* K96243, and then treated with 3 doses of 2.5 mg Novo at 1 h, 8 h, and 24 h post-challenge. The three control groups were vaccinated only, treated only, or were given PBS alone. The vaccinated mice received 2 doses of live vaccine via SC injection on day 0 and day 21, and the vaccine doses were 7.34 × 10^6^ and 5.92 × 10^6^ CFU, respectively. The mice were exposed to an inhaled dose of approximately 201 CFU of *B. pseudomallei* K96243 (8 LD_50_) 28 days later. All groups had 10 mice except the vaccinated, untreated group contained 9 mice.

Melioidosis is a notoriously difficult disease to prevent with prophylactic vaccination or to treat with post-exposure antibiotics. To explore the use of Novo in combination with a partially-protective live attenuated vaccine, we vaccinated BALB/c mice with the *B. thailandensis* E555 Δ*ilvI* vaccine strain. Vaccinated or sham-vaccinated mice were exposed to aerosolized *B. pseudomallei* K96243 (approximately 201 CFU or 8 LD_50_ equivalents). Treated mice were then given a 3-dose regimen of Novo (1 h, 8 h, and 24 h). The vaccinated mice that received Novo survived significantly longer than did the vaccinated mice or the sham-vaccinated mice receiving Novo alone (Figure 4). The survival rates on day 21 after challenge were 80% for the vaccinated mice treated with Novo compared to 0%, 11.1%, or 30% for the PBS controls, *B. thailandensis* E555 Δ*ilvI* vaccinated mice, and Novo treated mice (*p* = 0.0007, 0.0055, and 0.070, respectively). All of the mice succumbed to infection or were euthanized by day 51 post-infection. The geometric mean TTM of the treated and vaccinated mice was significantly longer than that of the other three groups, i.e., 35.1 days compared to 5.2 days (PBS), 13.0 days (vaccinated) and 15.1 days (Novo treated), with *p* < 0.0001, *p* = 0.016, and *p* = 0.040, respectively. Synergy analysis was performed as described below, and the findings revealed that combining antibiotic treatment with vaccination significantly synergized protection induced by the individual medical countermeasures in this model. The ratio of the fold increase in median survival time associated with vaccination in the antibiotic treated mice to the fold increase in survival time associated with vaccination in the absence of antibiotic treatment yielded a synergy score of 3.15 (*p* = 0.0060). BALB/c mice are a highly sensitive model of melioidosis; and the dose of aerosolized bacteria delivered in this study was greater than that depicted in Figure 3. Nevertheless, these findings support the strategy of layering partially protective vaccination strategies with sub-optimal antibiotic regimens to improve disease outcome, as reported recently (26). In future work, we will evaluate conditions which are conducive to significant protection and would allow a for the testing of delayed treatment regimens.

## 3. Discussion and Conclusions

These in vivo studies were initiated as an extension of our earlier in vitro studies with five bacterial biothreat agents [8]. The animal investigations with *B. pseudomallei* also expanded upon those described by Willcocks et al. [9]. In the latter, Novo and other aminocoumerins were evaluated for in vivo potency against *B. pseudomallei*. They were shown to be highly protective against lethal infection in the *Galleria* insect larvae model and in an intranasal challenge BALB/c mouse model when the mice were observed for 30 days post-challenge. In our studies, mice were infected by small-particle aerosol inhalation and survival was monitored for 60 days. Long-term tracking of survival better models the chronic nature of infections by the pathogenic *Burkholderia* and their potential for subclinical latency, relapse, and recrudescence [20,21]. Additionally, we characterized the effects of treatment on eradication (sterile immunity) of the infection in survivors. The current work also described the first investigation of the efficacy of Novo in a murine model of anthrax, where the antibiotic was shown to provide significant post-exposure protection against lethal infection. These results support the hypothesis presented in our previous in vitro sensitivity work and other studies that Novo and similar antibiotics exemplify currently licensed antibiotics which are capable of being repurposed as broadly effective initial treatments to back up the drugs-of-choice for highly pathogenic bacteria. In addition to *B. pseudomallei* and *B. anthracis*, the bacteria of major concern include the agents causing plague, glanders, and tularemia [8,12,39]. In our recent in vitro study, we used standard MIC susceptibility, direct killing, and time kill assays to evaluate the effectiveness of several rarely used licensed early generation antibiotics against these five bacterial biothreat agents. Novobiocin, tigecycline, and minocycline were identified as the most effective therapeutics against the pathogens. Low Novo MICs were obtained for *B. anthracis*, the *Burkholderia* pathogenic species, and *Francisella tularensis*. In agreement with previous reports, *Y. pestis* was novobiocin-resistant [39], although the inclusion of an antimicrobial peptide with Novo increased the in vitro susceptibility of *Y. pestis* [8].

Future efforts will focus on optimizing the Novo treatment regimen (number and timing of doses) and expanding on the strategy of combining vaccine pre-exposure prophylaxis and post-exposure antibiotic treatment. This layered strategy has the potential to enhance efficacy of medical countermeasures against bacterial pathogens refractory to antibiotic treatment and/or vaccine prophylaxis [8,40,41]. The significantly extended survival time after aerosol challenge with *B. pseudomallei* obtained by post-exposure treatment of mice vaccinated with a live vaccine strain provided further support of this approach (Figure 4).

In conclusion, the results of our murine studies advanced previous in vitro and in vivo findings by supporting the hypothesis that existing early-generation antibiotics, i.e., Novo and other aminocoumarins, can be repurposed as effective countermeasures against infection by a range of gram-positive and gram-negative bacterial biothreats. The protection provided by these licensed therapeutics might be enhanced by a novel platform combining pre-exposure prophylaxis and post-exposure treatment. Thus, continued efforts to optimize and increase therapeutic efficacy of aminocoumarin antibiotics against Tier 1 and other high consequence pathogens are warranted.

## 4. Materials and Methods

### 4.1. Bacteria, Media, and Growth Conditions

*B. pseudomallei* strain K96243 is a fully virulent strain that is often used to assess the efficacy of therapeutics and vaccines against melioidosis, and its preparation has been described in detail elsewhere [24,25]. In brief, a frozen stock aliquot of *B. pseudomallei* K96243 was grown in 4% glycerol (Sigma Aldrich, St. Louis, MO, USA) with 1% tryptone (Difco, Becton Dickinson, Sparks, MD, USA) and 5% NaCl (Sigma Aldrich) broth (GTB) at 37 °C with shaking until late log phase. For preparation of mouse challenge doses, the bacteria were harvested, resuspended in GTB, and quantified by OD_620_ estimation. The final delivered dose, described as the number of colony forming units (CFU), was then verified by plate counts on sheep blood agar (Trypticase soy agar with sheep blood) plates (Remel™, Thermo-Fisher Scientific, Waltham, MA, USA). *B. thailandensis* is a nonpathogenic environmental species of *Burkholderia*. Strain *B. thailandensis* E555 Δ*ilvI* was derived from *B. thailandensis* E555, a natural isolate that had genetically acquired the genes encoding the capsular polysaccharide expressed by the pathogenic *Burkholderia* species [42,43]. The deletion of the gene which encodes a subunit of an acetolactate synthase (*ilvI*) that is required for the synthesis of the branched chain amino acids isoleucine, leucine, and valine has been shown to greatly attenuate bacterial virulence, *e.g.,* this deletion in *B. pseudomallei* produced a mutant strain that was greatly weakened yet that effectively protected against lethal infection with *B. pseudomallei* [31,40,44]. The detailed DNA manipulation and cloning steps involved in creating *B. thailandensis* E555 Δ*ilvI* are described in Table S1 [42,43,45,46,47]. The *B. thailandensis* vaccine strain was streaked onto LB plates and the growth from the plates was used to inoculate LB Lennox broth(Difco). The overnight LB cultures were then diluted into PBS and administered to the mice.

Sterne is an attenuated strain of *B. anthracis* that produces the anthrax toxins but is unable to produce the anti-phagocytic capsule, a major virulence factor [3,4,5]. It is safe to handle and is widely used as a veterinary anthrax vaccine. Toxin-producing, nonencapsulated strains of *B. anthracis* cause disease resembling that due to fully virulent *B. anthracis* in strains of mice such as A/J, which are incapable of producing complement component C5a [5,43,48,49]. Stocks of purified ungerminated spores of *B. anthracis* strain Sterne were prepared as described elsewhere [48,49,50,51].

### 4.2. Animal Challenges and Monitoring

The strains of mice used were BALB/c, acquired from Charles River (Frederick, MD, USA), and A/J, from the Jackson Laboratory (Bar Harbor, ME, USA). The animal research was conducted under an animal use protocol approved by the USAMRIID Institutional Animal Care and Use Committee (IACUC) in compliance with the Animal Welfare Act, PHS Policy, and other Federal statutes and regulations relating to animals and experiments involving animals. The facility where this research was conducted is accredited by the AAALAC International and adheres to principles stated in the Guide for the Care and Use of Laboratory Animals [52].

Infection experiments with *B. anthracis* were performed with strain A/J mice inoculated by the subcutaneous (SC) or intranasal routes with purified ungerminated spores [48,53,54,55]. The A/J mouse and other mouse strains deficient in complement component C5 are highly susceptible to infection with attenuated unencapsulated toxin-producing strains of *B. anthracis* such as the Sterne strain. This animal model is widely used because of its safety and the production of a disease that resembles that due to fully virulent *B. anthracis* strains [5,49]. Before intranasal instillations, mice were anesthetized with ketamine, acepromazine, and xylazine injected IP. Doses given by SC routes were administered in a total volume of 200 µL. For intranasal challenges, a 50 µL inoculum of spores suspended in water for injection (Corning Cellgro, Corning, NY, USA) was placed on the nares for inhalation into the lungs [56].

For *B. pseudomallei* challenges, BALB/c mice were infected by the IP or the inhalational routes, as described previously [25,27]. BALB/c mice are a highly susceptible model for acute melioidosis [24,25,26,28]. Female mice which were 7 to 9 weeks of age at time of exposure were used in both models, in part due to the aggressive and injury-inducing behavior of males, which can confound results in long-duration studies such as those with *B. pseudomallei* [57]. Doses given by IP routes were administered in a total volume of 200 µL. For the exposures to aerosolized bacteria, mice were placed in a whole-body aerosol chamber within a Class III biological safety cabinet located inside a BSL-3 laboratory. Mice were then exposed to aerosols of *B. pseudomallei* suspension created by a three-jet collison nebulizer [27]. Samples were collected from the all-glass impinger vessel and analyzed bacteriologically to determine the inhaled dose of *B. pseudomallei* in CFU as described below.

Challenged mice were observed at least daily for 21 or 60 days (*B. anthracis* or *B. pseudomallei* infections, respectively) for clinical signs of illness, as described previously [24]. Early intervention endpoints were used during all studies and mice were euthanized when moribund, according to an endpoint score sheet. Animals were scored on a scale of 0–8: where 0–3 = no significant clinical signs (e.g., slightly ruffled fur); 4–7 = significant clinical symptoms such as subdued behavior, hunched appearance, absence of grooming, hind limb mobility and/or pyogranulomatous lesions of varying severity; ≥ 8 = distress. Those animals receiving a score of ≥8 were euthanized. Animals that survived were euthanized at the study endpoint; and in some studies, tissues were collected for bacteriological analyses.

### 4.3. Antibiotic Treatments and Vaccine

Novobiocin (Novo) was obtained from Sigma Aldrich (St. Louis, MO, USA), N6160—USP grade. Final concentrations of Novo were prepared in water for injection (Corning Cellgro, Corning, NY, USA) and were administered by the IP route in 200 µL volumes. Dosing regimens for Novo were determined based on previous murine model publications, such as the studies of Rodriguez-Cerrato et al. [58] and Marcu et al. [59], which provided PK, efficacy, and toxicity information; recommendations by our institute pharmacologist; and our preliminary Novo dose titration studies (described in the manuscript and data not shown). Strain *B. thailandensis* Δ*ilvI* was prepared in Gibco PBS (Thermo Fisher Scientific, Waltham, MA, USA) and injected SC, in a 200 µL volume, into the loose skin on the back of the mouse. The prime vaccination was approximately 7.4 × 10^6^ CFU and the booster delivered 28 days later was approximately 5.9 × 10^6^ CFU.

### 4.4. Bacteriology

The tissues collected from necropsied mice included lung and/or spleen, as indicated for each study. Organs were weighed, homogenized with disposable precision homogenizers (Covidien, Dublin, Republic of Ireland), and the CFU of the homogenate were determined on sheep blood agar plates. Undiluted homogenate and 10-fold dilutions in PBS (Dulbecco’s phosphate buffered saline, without Ca or Mg) were plated in duplicate to determine sterility. The limit of detection was approximately 10–100 CFU/mL blood (depending upon the experiment) or 5 CFU/organ.

### 4.5. Statistics

Survival curves of the vaccinated and control mice were estimated with the Kaplan-Meier method and were compared statistically using the log-rank test with Graph Pad Prism 9.0 (GraphPad Software, San Diego, CA, USA) and SAS version 9.4 (SAS Institute Inc., Cary, NC, USA). Significant differences in survival rates after virulent challenge were determined using the Fisher Exact test. The time-to-mortality (TTM; defined as mice succumbing to infection or mice that were euthanized in accordance with approved early-endpoint euthanasia criteria) values were compared with the log-rank test using SAS version 9.4. The potential synergy between antibiotic and vaccine was analyzed by forming a test of interaction in a log-logistic accelerated failure time model. The synergy score is the fold increase in survival time associated with vaccination in the antibiotic treated animals, divided by the fold increase in survival time associated with vaccination in the absence of antibiotic treatment. A Wald test was used to compare the synergy score to 1. Analysis was implemented in SAS version 9.4.

## Figures and Tables

**Table 1:** The bacterial burden in spleens from novobiocin-treated mice challenged with *B. pseudomallei*.

Novo Regimen	Mice Infected	Mice Survived	Sterile Mouse Spleens ^1^
2.5 mg 1 h	10	7	6 (86%)
2.5 mg 1 h, 8 h	10	8	8 (100%)
2.5 mg 1 h, 8 h, 24 h	10	8	8 (100%)
1.25 mg 1 h	10	1	1 (100%)
1.25 mg 1 h, 8 h	10	7	6 (86%)
1.25 mg 1 h, 8 h, 24 h	10	9	9 (100%)

^1^ limit of detection 5 CFU/spleen.

## Data Availability

The data presented in this study are available on request from the corresponding author.

## Supplementary Materials

The following supporting information can be downloaded at: https://www.mdpi.com/article/10.3390/antibiotics11121685/s1, Table S1: Strains, plasmids and primers used for construction of a *B. thailandensis* E555 Δ*ilvI* mutant.
