# Supplementary material for: Efficacy of Treatment with the Antibiotic Novobiocin against Infection with Bacillus anthracis or Burkholderia pseudomallei
Source: Antibiotics (Basel). 2022 Nov 23;11(12):1685. doi: 10.3390/antibiotics11121685 (PMC9774170; doi:10.3390/antibiotics11121685)
Supplement: Supplementary file 1 [file antibiotics-11-01685-s001.zip › antibiotics-1981069-supplementary.pdf]

antibiotics

MDPI

Supplementary File

Table S1. Strains, plasmids and primers used in construction of *B. thailandensis* E555 Δ*ilvI.*

Strain, plasmid or primer	Relevant characteristics*	Source or Reference
E. coli		
E. cloni®10G	General cloning and blue/white screening	Lucigen
SM10	Mobilizing strain with transfer genes of RP4 integrated on chromosome; Km^r	(30)
B. thailandensis		
E264	Soil isolate from central Thailand	(26)
E555	Cambodian soil isolate expressing a *B. pseudomallei*-like CPS gene cluster; Tp^s, Pm^r	(27)
E555 Δ*ilvI*	E555 harboring a 428-bp in-frame deletion in *ilvI*	This study
Plasmids		
pCR2.1-TOPO	3,931-bp TA vector; pMB1 *oriR*; Km^r, Ap^r	Life Technologies
pCR2.1-*Bt ilvI*	pCR2.1-TOPO harboring the 1,117-bp *Bt ilvI*-up and *Bt ilvI*-dn PCR product	This study
pCR2.1-Δ*Bt ilvI*	pCR2.1-*Bt ilvI* with a 428-bp *Sal*I fragment removed	This study
pEX18Tp-*pheS*	Gene replacement vector based on *pheS*; Tp^r	(31)
pEX18Tp-Δ*Bt ilvI*	pEX18Tp-*pheS* containing the *Nhe*I insert from pCR2.1-*ΔBt ilvI*	This study
Primers (5′-3′)		
Bt ilvI-up	GCTAGCCGCTCCTCTTGTCCGCGAAG	eurofins
Bt ilvI-dn	GCTAGCTACAGATCTTCCGATCCGAG	eurofins

* r, resistant; s, susceptible; Tp, trimethoprim; Pm, polymyxin B; Km, kanamycin; Ap, ampicillin.

Construction of *B. thailandensis* E555 Δ*ilvI* –

The methods used for DNA manipulation and PCR amplification have been described in detail elsewhere [29]. Gene replacement with *B. thailandensis* E555 was performed using the *pheS*-based vector pEX18Tp-*pheS*, as previously described [31]. A 1.1-kb portion of the *Burkholderia thailandensis* E264 *ilvI* gene, locus tag *BTH_I1045*, was PCR-amplified using purified genomic DNA and the primers *Bt ilvI*-up and *Bt ilvI*-dn (Table S1). The PCR product was cloned into pCR2.1-TOPO and a 428-bp *Sal*I fragment was removed and it was re-ligated. The resulting plasmid, pCR2.1-Δ*Bt ilvI*, was digested with *Nhe*I and the product was cloned into the *Xba*I site of pEX18Tp-*pheS* (Table S1). Plasmid pEX18Tp-*ΔBt ilvI* (Table S1) was electroporated into *Escherichia coli* SM10 (12.25 kV/cm) and conjugated with *B. thailandensis* E555 for 8 h on LB agar. Trimethoprim (Tp) was used at 300 µg/ml to select for merodiploid derivatives harboring the recombinant plasmid and polymyxin B (Pm) was used at 25 µg/ml to counterselect *E. coli* SM10. Multiple Tp^r colonies were inoculated onto M9 Minimal Salts agar plates with 0.4% glucose (M9), 0.1% (wt/vol) p-chlorophenylalanine, 40 µg/ml L-isoleucine, 40 µg/ml L-leucine and 40 µg/ml L-valine (ILV) and incubated at 37°C for 2 days. Individual colonies that grew on this medium were subsequently picked to LB agar and M9 agar plates and incubated at 37°C for 3 days. *B. thailandensis* E555 Δ*ilvI* was identified as not being able to grow on M9 agar without added ILV and by colony PCR using the primers *Bt ilvI*-up and *Bt ilvI*-dn (Table S1). As expected, the PCR product generated from *B. thailandensis* E555 Δ*ilvI* was smaller than the product from *B. thailandensis* E555 (data not shown).
